# A novel gene, *MdSSK1*, as a component of the SCF complex rather than *MdSBP1* can mediate the ubiquitination of *S*-RNase in apple

**DOI:** 10.1093/jxb/eru164

**Published:** 2014-04-23

**Authors:** Hui Yuan, Dong Meng, Zhaoyu Gu, Wei Li, Aide Wang, Qing Yang, Yuandi Zhu, Tianzhong Li

**Affiliations:** ^1^Laboratory of Fruit Cell and Molecular Breeding, College of Agronomy and Bio-tech, China Agricultural University, Beijing 100193, China; ^2^College of Horticulture, Shenyang Agricultural University, Shenyang 110866, China

**Keywords:** Apple, SCF complex, self-incompatibility, *S*-RNase, SSK, ubiquitin.

## Abstract

As a core factor in *S*-RNase-based gametophytic self-incompatibility (GSI), the SCF (SKP1–Cullin1–F-box-Rbx1) complex (including pollen determinant SLF, S-locus-F-box) functions as an E3 ubiquitin ligase on non-self *S*-RNase. The SCF complex is formed by SKP1 bridging between SLF, CUL1, and Rbx1; however, it is not known whether an SCF complex lacking SKP1 can mediate the ubiquitination of *S*-RNase. Three SKP1-like genes from pollen were cloned based on the structural features of the SLF-interacting-SKP1-like (SSK) gene and the ‘Golden Delicious’ apple genome. These genes have a motif of five amino acids following the standard ‘WAFE’ at the C terminal and, in addition, contain eight sheets and two helices. All three genes were expressed exclusively in pollen. In the yeast two-hybrid and pull-down assays only one was found to interact with MdSFBB and MdCUL1, suggesting it is the SLF-interacting SKP1-like gene in apple which was named *MdSSK1*. *In vitro* experiments using MdSSK1, S2-MdSFBB1 (S2-*Malus domestica* S-locus-F-box brother) and MdCUL1 proteins incubated with *S*_2_-RNase and ubiquitin revealed that the SCF complex ubiquitinylates *S*-RNase *in vitro*, while MdSBP1 (*Malus domestica S*-RNase binding protein 1) could not functionally replace MdSSK1 in the SCF complex in ubiquitinylating *S*-RNase. According to the above experiments, MdSBP1 is probably the only factor responsible for recognition with *S-*RNase, while not a component of the SCF complex, and an SCF complex containing *MdSSK1* is required for mediating the ubiquitination of *S-*RNase.

## Summary

The related components of the SCF complex in apple were cloned and it was proved that an SCF complex containing *MdSSK1* rather than *MdSBP1* can mediate the ubiquitination of *S*-RNase.

## Introduction

Self-incompatibility is a widespread barrier to reproduction in flowering plants that can prevent inbreeding and promote outcrossing by rejecting self (genetically related) pollen while leaving non-self (genetically unrelated) pollen for fertilization (De Nettancourt, 2001; Franklin-Tong, 2008). *S*-RNase-based gametophytic self-incompatibility (GSI) has been found in the Solanaceae, Rosaceae, and Plantaginaceae families (Lai *et al.*, 2002; Sijacic *et al.*, 2004), with ubiquitination of non-self *S*-RNase by the SCF (SKP1–Cullin1–F-box-Rbx1) complex a core factor in this process (Hua *et al.*, 2008). The SCF complex consists of SKP1 (S-phase kinase-associated protein 1), F-box, CUL1, and Rbx1 (Moon *et al.*, 2004). SKP1 connects F-box and CUL1, while the F-box interacts with *S*-RNase specifically, leading to ubiquitin degradation of *S*-RNase (Tyers and Jorgensen, 2000).

Comparison of the SSK (SLF-interacting-SKP1-like) genes reported in GSI plants revealed that they retain the 5–9 amino acid tail following the C terminus WAFE, besides the eight sheets and two helices (Gagne *et al.*, 2002; Risseeuw *et al.*, 2003; Huang *et al.*, 2006; Yang *et al.*, 2006; Chang *et al.*, 2009). The canonical SKP1 protein can be divided into two major domains: the N-terminal section that interacts with CUL1, and the C-terminal that combines with the F-box. Further, SSKs have numerous amino acid residue substitutions in the H5–H8 region (Schulman *et al.*, 2000; Zheng *et al.*, 2002). These changes may contribute to its specific affinity with SLF compared with other F-box proteins (Gray *et al.*, 1999; Samach *et al.*, 1999; McGinnis *et al.*, 2003). These SSKs are thought to interact with SLF and CUL1 to form the SCF complex which participates in the ubiquitin reaction of *S*-RNase. There have been some previous reports about SSK in plants such as petunia, antirrhinum, pear, and sweet cherry using GSI. The SSKs are very highly conserved, having no *S* haplotype specificity and are not linked to the *S*-locus, but they are expressed specifically in pollen (Huang *et al.*, 2006; Zhao *et al.*, 2010; Matsumoto *et al.*, 2012; Xu *et al.*, 2013). Current studies suggest that SSK can interact with SLF and CUL1 *in vitro*, and can also bind with *S*-RNase via SLF in pollen extracts (Huang *et al.*, 2006; Zhao *et al.*, 2010). In petunia, some research has indicated that the SCF complex contains SLF, Cullin1, SSK1, and RBX1 (Li *et al.*, 2014), but the component of the SCF complex in apple which causes the ubiquitination of *S*-RNase is still unknown.

Further, as a RING-HC protein PiSBP1 (*Petunia inflata S*-RNase binding protein 1) can interact with the hypervariable region of *S*-RNase; the former is considered to adopt the function of SKP1 and Rbx1 in bridging CUL1 and the F-box, forming the SCF complex (Hua and Kao, 2006). PiSBP1 is known to function as an E3 ubiquitin ligase in recognizing and degrading *S*-RNase *in vitro* (Hua and Kao, 2008). In petunia, both SBP1 and SSK1 were inferred to be part of the SCF complex which degrades *S*-RNase (Sims *et al.*, 2010), but recent research has indicated that PiSBP1 is not included in the SCF complex (Li *et al.*, 2014). However, in rosaceous plants there have been no related reports.

The components of the apple SCF complex (SSK, SFBB, CUL1, and SBP1) were cloned based on the apple genome and the EST database. Their interactions were examined using the yeast two-hybrid and pull-down assays which identified the candidates SSK1, SFBB, CUL1, and SBP1. The results revealed that an SCF complex containing SSK1 rather than SBP1 can mediate the ubiquitination of *S*-RNase.

## Materials and methods

### Plant materials

Samples of apple (*Malus domestica* Borkh.) were taken from the Liaoning Institute of Pomology. Leaves, pollen, pistils, petals, sepals, and ovaries were collected in the spring, frozen in liquid nitrogen, and stored at –80 °C for later use. The 16 cultivars were: ‘Golden Delicious’ (*S*_2_ *S*_3_), ‘Ralls Janet’ (*S*_1_ *S*_2_), ‘Fuji’ (*S*_1_ *S*_9_), ‘Red Chief’ (*S*_9_ *S*_19_), ‘Gala’ (*S*_2_ *S*_5_), ‘Maypole’ (*S*_10_ *S*_16_), ‘Jiguan’ (*S*_3_ *S*_5_), ‘Jonathan’ (*S*_7_ *S*_9_), ‘Qiyuexian’ (*S*_1_ *S*_19_), ‘Megumi’ (*S*_2_ *S*_9_), ‘Sansa’ (*S*_5_ *S*_7_), ‘Tianhuangkui’ (*S*_5_ *S*_22_), ‘Granny Smith’ (*S*_3_ *S*_23_), ‘Tsugaru’ (*S*_3_ *S*_7_), ‘Liaofu’ (*S*_1_ *S*_20_), and ‘Tuscan0’ (*S*_3_ *S*_10_).

### RT-PCR and the analysis of gene expression patterns

Genomic DNA was extracted from leaves using the CTAB method. The total RNA of pollen, styles, ovaries, petals, sepals, and leaves was isolated using a slightly modified CTAB method (Chang *et al.*, 1993), and digested with DNase I (TaKaRa). cDNA was synthesized by SuperScript III (TaKaRa) and a poly-dT primer. RT-PCR was used to analyse the expression patterns of the genes. As a control, the apple actin gene (AB638619.1) was also used. The primers for each gene are listed in Supplementary Table S1 at *JXB* online.

### Yeast two-hybrid assays

The CDS for *MdSBP1*, *MdSSK1*, two *MdSKPL*s, and six S-RNases were cloned into pGBKT7 (Clontech, CA, USA). Meanwhile, the CDS for *MdCUL1* and 10 *MdSFB*s were cloned into pGADT7 (Clontech, CA, USA). Different BD and AD vectors were co-transformed into AH109 and grown on SD/-Leu-Trp medium at 30 °C for 3–4 d. Ten different clones for each combination were grown on SD/-Ade-His-Leu-Trp medium at 30 °C for 3–4 d and then stained with X-a-gal (Clontech) to detect their interaction.

### Pull-down assays

The coding regions of *MdSSK1* and *MdSBP1* were cloned into the pGEX-4T-1 vector (CW biotech) to produce GST fusion proteins. The CDSs of *MdCUL1*, *S2*-*MdSFBB1*, *S2*-*MdSFBB2*, *S2*-*MdSFBB3*, and *S2*-*MdSFBB4* were cloned into the pMAL-c2X (NEB) vector to produce MBP fusion proteins. All the vectors were transformed into *E. coli* strain BL21 or BL21 DE3 (Transgen). Cells were cultured in LB medium containing 50 µg ml^–1^ ampicillin at 37 °C with shaking at 200rpm. When the OD_600_ achieved 0.6, IPTG was added to the suspension culture to induce the expression of proteins of interest. The induction conditions were 23 °C for 12–6h and 0.2 mmol IPTG.

Centrifugation was performed in order to gather bacteria and then a PBS buffer (0.137mol l^–1^ NaCl, 0.0027mol l^–1^ KCl, 0.01mol l^–1^ Na_2_HPO_4_, 0.002mol l^–1^ H_2_PO_4_, pH 7.8) was used to suspend the bacteria. Next, an ultrasonic cell disruptor was used at 200W for crushing; the ultrasonic frequency worked for 10 s, resting for 5 s for 25min. Finally, the material was centrifuged at 10 000rpm for 1h. The MBP fusion proteins were purified by amylase resin (NEB). After washing twice by column buffer (0.02mol l^–1^ TRIS–HCl pH 7.4, 0.2mol l^–1^ NaCl, 0.01mol l^–1^ β-mercaptoethanol, 0.001mol l^–1^ EDTA buffer), the proteins were eluted by 500 µl elution buffer (column buffer with 0.01mol l^–1^ maltose). The GST fusion proteins were purified by glutathione sepharose 4B beads and were eluted by GSH elution buffer (0.061g glutathlone, 500 µl 1mol l^–1^ TRIS–HCl pH 8.0 in 10 µl buffer). All these processes were performed at 4 °C.

For the pull-down assay, purified MBP-MdCUL1 protein was adsorbed on to amylase resin (NEB), then an equivalent amount of purified GST-MdSSK1 protein was added to the column. After incubation at 4 °C for 1h, the column was washed twice with PBS buffer in order to clear away unbound proteins. Next, bound proteins were eluted with elution buffer. The eluted proteins were boiled for 5min, separated by 12% SDS-PAGE, transferred on to NC membrane (CW Biotech), and probed with anti-GST. GST-tag protein and MBP-tag protein were used as negative controls. For other pull-down assays, the procedure was performed in a similar way.

### Ubiquitin analysis of *S*-RNase

His-tagged *S*-RNase was purified with Ni-NTA resin. 50mg pollen was dissolved in 2.5ml pollen culture medium, containing 10g sucrose, 0.01g boric acid, and 0.015g calcium chloride. It was allowed to stand for 10min at room temperature, after which different concentrations of *S*-RNase were added to the medium, as indicated in Supplementary Fig. S6 at *JXB* online. They were placed at 23 °C for 2h in order to let the pollen tube elongate. The germination rate and pollen tube length were then calculated. Afterwards 1ml fixing buffer was added to fix the pollen tube and it was centrifuged at 200rpm for 5min. Then proteins were extracted from the pollen tube using the Applygen protein extraction kit and Western blotting was performed to detect *S*-RNase.

*In vitro* ubiquitination assays were performed as described elsewhere (Yang *et al.*, 2009; Zhao *et al.*, 2012). *S*-RNase was ubiquitinated in a reaction mixture containing 50mM TRIS (pH 7.4), 10mM MgCl_2_, 2mM DTT, 5mM HEPES, 2mM ATP, 0.05% Triton X-100, 10mM creatine phosphate, 1µ phosphokinase, 10 µg ubiquitin, 50nM E1 (UBA6, *Petunia hybrida*), 1mM PMSF, 850nM E2 (UBH6, *Petunia hybrida*), GST-MdSSK1, MBP-MdCUL1, and MBP-S_2_-MdSFBB1 at 30 °C for 2h. Mixtures were immunoblotted with anti-*S*-RNase.

### Sequence analysis

The gene analysis and chromosomal locations were searched using the apple genome database (http://genomics.research.iasma.it/, http://linux1.softberry.com/berry.phtml). Genes belonged to the SKP1 family were used to construct phylogenetic trees（http://www.ncbi.nlm.nih.gov/). The deduced amino acid sequences of the SKP1 family was used to generate a phylogenetic tree using the NJ method with 1 000 bootstrap replicates using MEGA version 5.0 (Tamura *et al.*, 2011).

## Results

### Cloning and expression analysis of apple *SKP1-like*, *SFBB*, and *CUL1* genes

**Fig. 1.**
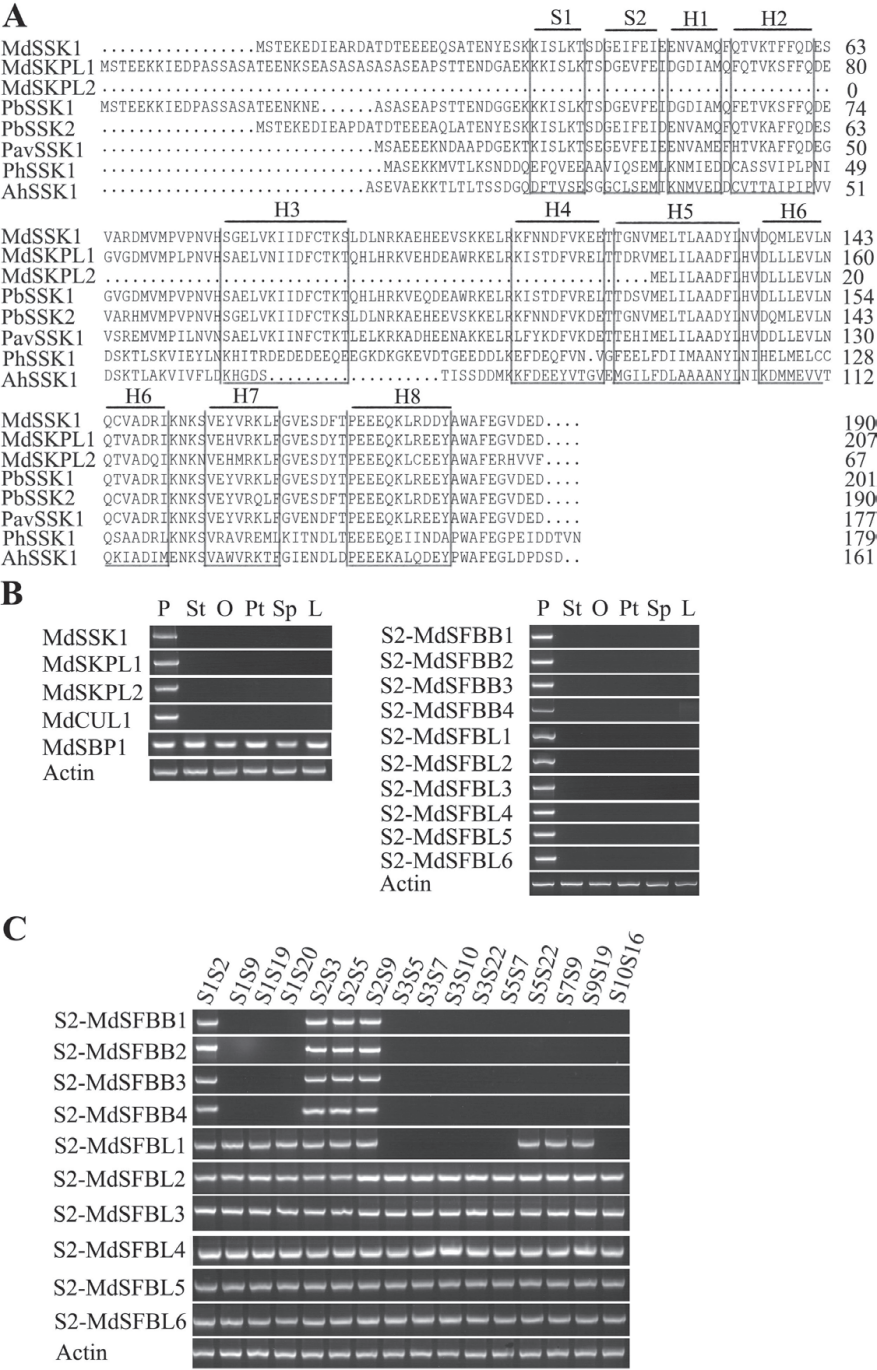
Sequence alignment of SSK genes and the expression patterns of the genes in this research. (A) Sequence alignment and structural elements of SSKs. PavSSK1 (JQ322646), PhSSK1 (FJ490176), AhSSK1 (DQ355479), PbSSK1 (HE802072), PbSSK2 (HE802071), MdSSK1, MdSKPL1, and MdSKPL2 are from this study. The boxes represent the secondary structure. *S* β-sheet, *H* α-helix. (B) The expression patterns of MdSSK1, MdSKPL1, MdSKPL2, MdCUL1, MdSBP1, *S2*-MdSFBB1–4, and *S2*-MdSFBL1–6 were examined by RT-PCR. Total RNAs of different organs were extracted and used as templates for cDNA synthesis and RT-PCR. L (leaves), Sp (sepals), Pt (petals), O (ovaries), St (styles), P (pollen). (C) The haplotype specificity expression patterns of S2-MdSFBB1–4 and *S2*-MdSFBL1–6 were examined by RT-PCR. Total DNAs of 16 cultivars were extracted and used as templates for RT-PCR.

Using the conserved amino acid of the C terminal domain of *SSK* in petunia, antirrhinum, pear, and sweet cherry, a Blast search of the apple genome was performed (http://genomics.research.iasma.it/) (Velasco *et al.*, 2010) and then the contig was annotated using Softberry (http://linuxl.softberry.com/berry.phtml). Three candidate SSK genes were obtained. Specific primers were designed and the full length of the three candidate SSK genes was obtained using ‘Golden Delicious’ (*S*_2_ *S*_3_) pollen cDNA as a template. The amino acid sequence identities between the three genes and other SSKs in petunia, antirrhinum, pear, and sweet cherry were 56.03%, 57.72%, and 46.18% (Fig. 1A), respectively. The three SKP1-like genes were clustered with the *SSK*s in pear and sweet cherry (see Supplementary Fig. S1 at *JXB* online). Two of the SKP1-like genes contain eight sheets and two helices and have a shared sequence of five amino acids following the WAFE motif, whereas the third contained only the C terminal domain and the WAFE motif, and lacked ~140 amino acids at the N terminal. The WAFE motif differed from previous reports (Fig. 1A), the latter described as WAFEGVDED in pear and sweet cherry，while the third SKP1-like gene contained WAFERHVVF. RT-PCR analysis showed the three genes were all expressed specifically in pollen (Fig. 1B).

**Fig. 2.**
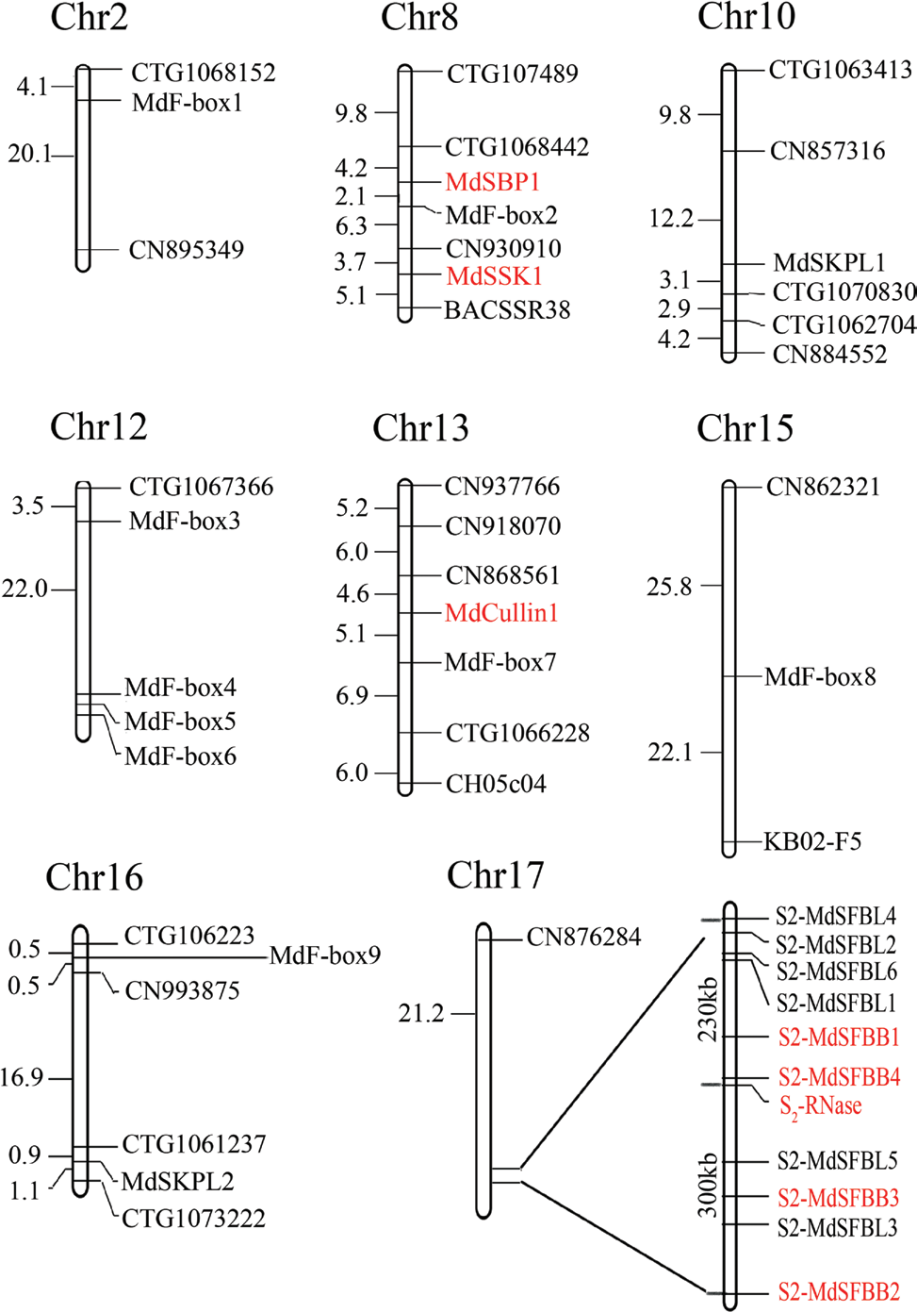
The genomic location of apple genes used in this research. MdSBP1, MdSSK1, MdCullin1, *S*_2_-RNase and the four SFBBs that have haplotype specificity are marked in bold. (This figure is available in colour at *JXB* online.)

Nineteen genes containing an F-box motif at the N terminal were found by a Blast search of the ‘Golden Delicious’ genome using the conserved F-box domain of SLF as query. These genes were located on chromosomes 2, 8, 10, 12, 15, 16, and 17 (Fig. 2). Ten F-box genes were located on chromosome 17, on which the *S*-locus was located. Of these, six were located upstream of *S*_*2*_ *-RNase*，the furthest distance being 230kb, and four were downstream no further than 300kb (Fig. 2). The similarity of the 10 genes was 56.38% and cluster with other *SFBB*s (see Supplementary Fig. S2 at *JXB* online). The pollen cDNA was used as a template for cloning the 19 *F-box* genes, and the sequences were found to be identical to those obtained from the apple genome. Expression analysis determined that the 10 genes on chromosome 17 were expressed specifically in pollen (Fig. 1B), while the remaining nine genes from the other chromosome did not have tissue specificity (see Supplementary Fig. S3 at *JXB* online). The expression patterns of the 10 genes were analysed in 16 different apple cultivars and it was found that four genes were expressed specifically in the cultivar containing the *S*_2_ haplotype (Fig. 1C). According to the expression patterns and in order to distinguish them with other SFB genes that had been reported previously (Minamikawa *et al.*, 2010; Kakui *et al.*, 2011), the four genes that had haplotype specificity were considered to be the SFBB gene (S-haplotype-specific F-box) and named them *S2-MdSFBB1* (*S*-locus F-box brother1), *S2-MdSFBB2*, *S2-MdSFBB3*, and *S2-MdSFBB4*, respectively; the other six genes on the 17th chromosome *S2-MdSFBL1* (*S*-locus F-box like1), *S2-MdSFBL2*, *S2-MdSFBL3*, *S2-MdSFBL4*, *S2-MdSFBL5*, and *S2-MdSFBL6*; and the nine genes located on the other chromosomes were named *MdF-box1–9*.

Meanwhile, a gene with high amino acid sequence identity (78.54%) to *PbCUL1* was noted in the apple genome. The cDNA of ‘Golden Delicious’ was used as the template for cloning this gene. It encoded 772 amino acids and clustered with *Arabidopsis* AtCUL1 (see Supplementary Fig. S4 at *JXB* online) which is one component of the SCF complex (Marin, 2009). It was found to be expressed specifically in pollen (Fig. 1B) and was assigned the name *MdCUL1*.

The chromosome location of the genes used in this paper were done based on markers supplied by Han *et al.* (2011).

### Yeast two hybrid (Y2H) assay and pull-down analysis of MdSKP1-like, MdSFB, and MdCUL1

**Fig. 3.**
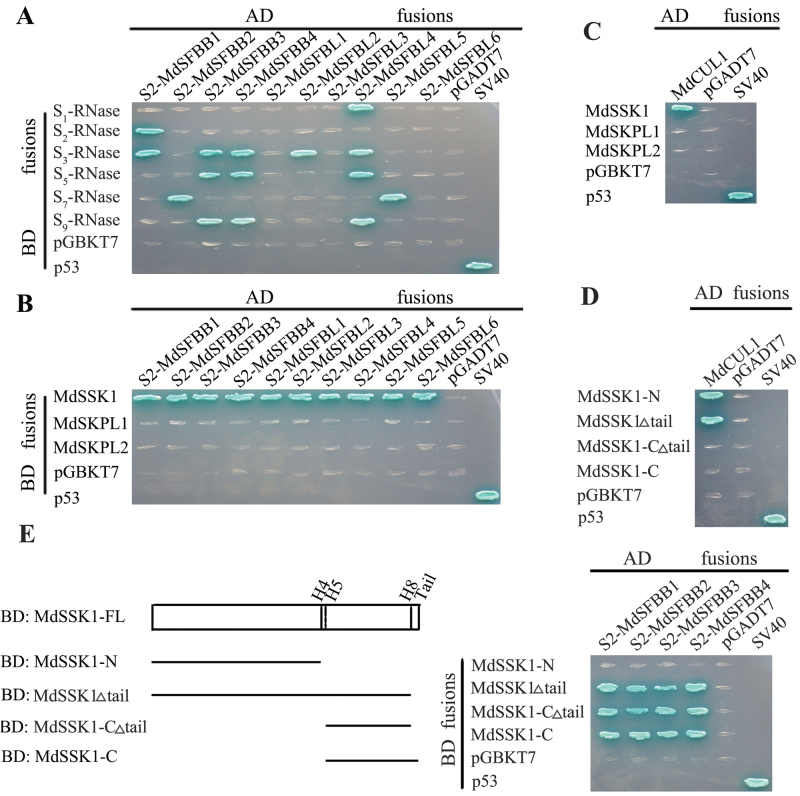
Yeast two-hybrid (Y2H) analysis of MdSKP1-like, MdSFB, and MdCUL1. (A) Y2H assays of S2-MdSFBB1–4 and S2-MdSFBL1–6 with *S*-RNases.(B) Y2H assays of MdSSK1, MdSKPL1–2 with S2-MdSFBB1–4 and S2-MdSFBL1–6. (C) Y2H assays between MdSSK1, MdSKPL1–2, and MdCUL1. (D) The interaction between different parts of MdSSK1 and MdCUL1. (E) The left indicates the structure that every vector represents. The right shows the interaction between different parts of MdSSK1 and S2-MdSFBB1–4. The AD fusions and BD fusions were co-transformed into yeast strain AH109. Transformants were grown on selective medium SD/-Ade-His-Leu-Trp for 7 d at 30 ° and then were stained by X-α-gal. The p53 and SV40 were used as positive controls, the pGBKT7 and pGADT7 as negative controls. (This figure is available in colour at *JXB* online.)

The full-length coding sequences of pistil *S*_*1*_ *-*, *S*_*2*_ *-*, *S*_*3*_ *-*, *S*_*5*_ *-*, *S*_*7*_ *-*, and *S*_*9*_ *-RNase* without signal peptides (S-mat) were placed into the pGBKT7 vector and *S2-MdSFBB1–4* and *S2-MdSFBL1–6* were placed into the pGADT7 vector and they were co-transformed into AH109. The results indicated that S2-MdSFBB1 could interact with *S*_*2*_ *-*, *S*_*3*_-RNase, S2-MdSFBB2 could interact with *S*_*7*_-RNase, while S2-MdSFBB3 and S2-MdSFBB4 could bind with *S*_*3*_ *-*, *S*_*5*_ *-*, and *S*_*9*_-RNase (Fig. 3A). A non-S-RNase was used as a control, the results indicated that the non-S-RNase could not interact with S2-MdSFBB1-4 in yeast (see Supplementary Fig. S5 at *JXB* online). A non-S-RNase belongs to the RNase family, it has the same structure but is not located on the S-locus.

Each of these three MdSKP1-like genes were cloned into the pGBKT7 vector, and *MdCUL1* was placed into the pGADT7 vector. Only a single MdSKP1-like gene, named *MdSSK1*, was found to interact with the 10 MdSFB genes and MdCUL1 in Y2H (Fig. 3B, C). The remaining two were named *MdSKPL1* (MdSKP-like1) and *MdSKPL2* (MdSKP-like2). Then MdSSK1 was divided into four parts and they were cloned into the pGBKT7 vector (Fig. 3E), the results indicated that the BD vector that lacked the N terminal of MdSSK1 could not interact with MdCUL1 (Fig. 3D), while the one lacking the C terminal could not bind with the MdSFBB (Fig. 3E).

**Fig. 4.**
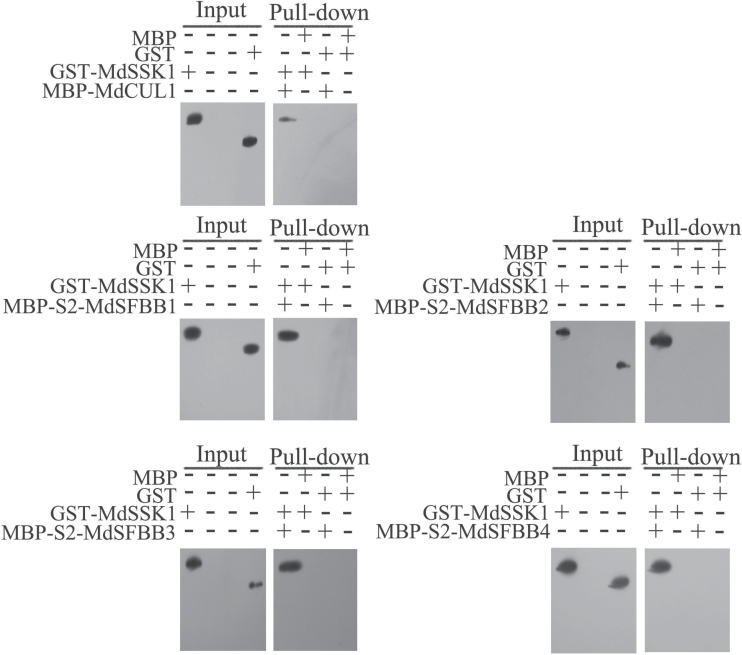
Pull-down analysis between MdSSK1 and MdCUL1 and S2-MdSFBB1–4. Purified MBP-MdCUL1 and MBP-S2-MdSFBB1–4 were used as bait against purified GST-MdSSK1. Bound proteins were examined with anti-GST antibody. GST and MBP were used as negative controls.

Next, *MdSSK1* was transformed into the prokaryotic expression vector pGEX-4T-1 and *MdCUL1*, *S*_*2*_ *-MdSFBB1*, *S*_*2*_ *-MdSFBB2*, *S*_*2*_ *-MdSFBB3*, and *S*_*2*_ *-MdSFBB4* into the pMAL-c2X vector. Pull-down analysis showed that GST-MdSSK1 could combine with MBP-MdCUL1, MBP-*S*_*2*_-MdSFBB1, MBP-*S*_*2*_-MdSFBB2, MBP-*S*_*2*_-MdSFBB3, and MBP-*S*_*2*_-MdSFBB4 (Fig. 4).

### Yeast two-hybrid and pull-down assay between MdSBP1 and MdSSK1, MdSFB, MdCUL1, *S*-RNase

Based on the conserved domain of PiSBP1 in petunia, a Blast search of the ‘Golden Delicious’ apple genome was performed and the homologue *MdSBP1* was retrieved. This gene was cloned and compared with the other SBP1 proteins. The results indicated that MdSBP1 is a RING-HC finger protein containing eight conserved cysteines at the C terminal, as with PiSBP1 and NaSBP1 in petunia and nicotiana (see Supplementary Fig. S6A at *JXB* online). The expression pattern of this gene had no specificity; it was expressed in pollen, style, leaves, and other organs examined (Fig. 1B).

**Fig. 5.**
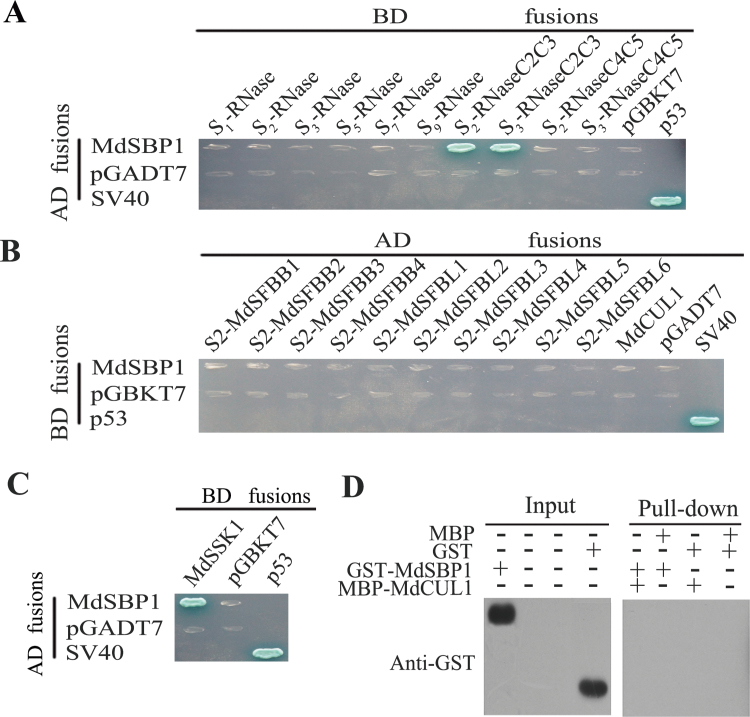
Identification of the SBP1 protein in apple. (A) Y2H analysis between MdSBP1 and different haplotype of *S*-RNases. *S*_1_-, *S*_2_-, *S*_3_-, *S*_5_-, *S*_7_-, and *S*_9_-RNase represent the full length, while *S*-RNase-C2HVC3 and *S*-RNaseC4C5 represent the hypervariable and conserved region, respectively. The detail of this is in Supplementary Fig S7 at *JXB* online. (B) Y2H analysis between MdSBP1 and MdCUL1, S2-MdSFBB1–4, and S2-MdSFBL1–6. (C) The interaction analysis between MdSBP1 and MdSSK1. The AD fusions and BD fusions were co-transformed into yeast strain AH109. Transformants were grown on selective medium SD/-Ade-His-Leu-Trp for 7 d at 30 ° and then were stained by X-α-gal. The p53 and SV40 were used as positive controls, the pGBKT7 and pGADT7 as negative controls. (D) Pull-down analysis of MdSBP1 and MdCUL1. Purified MBP-MdCUL1 was used as bait against purified GST-MdSBP1. Bound proteins were examined with anti-GST antibody. GST and MBP were used as negative controls.

Apart from interacting with the hypervariable region of the *S*_*2*_, *S*_*3*_-RNase (Fig. 5A), the yeast two-hybrid assay showed that MdSBP1 could also interact with MdSSK1 (Fig. 5C), although it did not combine with the full-length sequence of *S*-RNase, MdSFBB, and MdCUL1 (Fig. 5A, B, D; see Supplementary Fig. S5B at *JXB* online).

### *S*-RNase is ubiquitinated by a constructed SCF complex containing MdSSK1 *in vitro*

*S*-RNase is taken up into pollen tubes and ubiquitinated in compatible pollination in *Antirrhinum* (Qiao *et al.*, 2004). In order to detect whether *S*-RNase could be ubiquitinated in apple, the changes of *S*-RNase *in vitro* and semi-*in vivo* were analysed.

First, bud stage flowers of ‘Golden Delicious’ (*S*_*2*_ *S*_*3*_) were collected and treated with different concentrations of His-tag *S*_*2*_-RNase. The germination rate and pollen tube length were observed, and it was found that 25 µg ml^–1^ was the suitable concentration for culturing pollen (see Supplementary Fig. S7 at *JXB* online).The results indicated that S-RNase can be taken up into the pollen tubes and influence pollen tube growth.

**Fig. 6.**
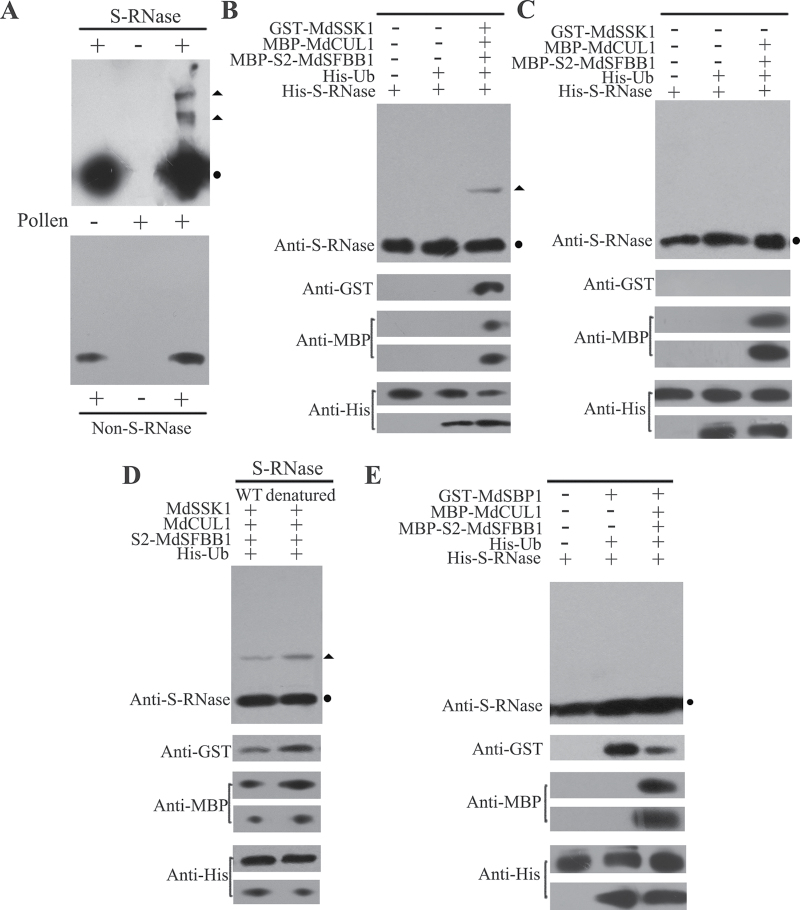
Detection of ubiquitinated *S*-RNases. (A) The pollen tube total proteins after incubation with purified *S*-RNase were detected with anti-S-RNase antibody by Western blot. The non-*S*-RNase was used as a control. The ubiquitination of *S*-RNase was analysed in the presence of MdSSK1, MdCUL1, S2-MdSFBB1, and His-Ub (B) or without MdSSK1 (C); reactions containing His-Ub or His-Ub and *S*-RNase were carried out as controls. (D) The ubiquitination of denatured *S*-RNase were also detected. The circles indicate the predicted size of *S*-RNases, the triangles indicate the ubiquitinated form of *S*-RNases. (E) The ubiquitination of *S*-RNase was analysed in the presence of MdSBP1, MdCUL1, and S2-MdSFBB1; reactions containing His-Ub or His-Ub and *S*-RNase were carried out as controls. By anti-MBP examination, the upper bands indicated MBP-*S*2-MdSFBB1, below that for MBP-MdCUL1; for anti-His, the upper and lower bands indicated His-Ub and His-*S*-RNase, respectively in (B), (C), (D), and (E). (This figure is available in colour at *JXB* online.)

It was then analysed whether there were any changes in the *S*-RNase that was taken up into the pollen tubes. Total protein of the pollen tubes was extracted using the applygen protein extraction kit after being treated with 25 µg ml^–1^ *S*_*2*_-RNase for 2h, followed by the detection of changes in *S*_*2*_-RNase using anti-*S*_*2*_-RNase. The results showed two discrete bands with molecular masses of 66kDa and 86kDa, which were higher than that of His-*S*_*2*_-RNase (approximately 26kDa), While using non-*S*-RNase as a control, no band could be detected except the non-*S*-RNase (Fig. 6A). These two discrete bands were thought to be the ubiquitinated form of His-*S*_*2*_-RNase.

So, in order to test whether S-RNase can be ubiquitinated *in vitro*, His-UBA6 was used as the E1, His-UBH6 as the E2, then purified MdSSK1, S2-MdSFBB1, and MdCUL1 as E3. E1, E2, and E3 were mixed together and *S*_2_-RNase and His-Ub were added to the mixture and co-incubated for 2h (Stone *et al.*, 2003; Hua and Kao, 2008; Yang *et al.*, 2009; Zhao *et al.*, 2012). An anti-*S*_*2*_-RNase antibody was used to detect the ubiquitinated His-*S*_*2*_-RNase by Western blot. A distinct band with higher molecular mass (about 66kDa) than His-*S*_*2*_-RNase was detected; the band was not detected in the reactions where only *S*_*2*_-RNase or *S*_*2*_-RNase and His-Ub were added (Fig. 6B), and neither in the reaction omiting MdSSK1 (Fig. 6C). A band with molecular mass higher than His-*S*_2_-RNase was also detected in the reaction with denatured *S*_*2*_-RNase included (Fig. 6D), indicating that the denatured *S*-RNase can also be ubiquitinated *in vitro*.

No ubiquitinated band of *S*-RNase was detected in the reaction in which MdSSK1 was replaced by MdSBP1 (Fig. 6E). There was also no ubiquitinated form of *S*-RNase in the reaction that only contained MdSBP1 (Fig. 6E).

## Discussion

As a key component of the SCF complex, pollen *SSK* is integral in *S*-RNase-based gametophytic self-incompatibility. This protein bridges F-box and CUL1, forming the SCF complex, and participates in the degradation of non-self *S*-RNase while leaving self *S*-RNase active to function as a cytotoxin (Sijacic *et al.*, 2004; Hua *et al.*, 2008). Thus far, there have been many reports that SSK can interact with corresponding SLF and CUL1 in petunia, antirrhinum, pear, and sweet cherry (Huang *et al.*, 2006; Zhao *et al.*, 2010; Matsumoto *et al.*, 2012; Xu *et al.*, 2013). The co-incubation of style extracts and pollen extracts indicated that the *S*-RNase in the style can be ubiquitinated by certain factors in the pollen extracts in antirrhinum (Qiao *et al.*, 2004). Both GST-*S*_1_-RNase and GST-*S*_2_-RNase when co-incubated with the pollen tube extracts of *S*_2_ homozygotes can be ubiquitinated, and GST-*S*_3_-RNase can also be ubiquitinated by the pollen tube extracts of *S*_2_ homozygotes. These observations indicate that ubiquitination ‘semi *in-vivo*’ is not *S*-specific (Hua and Kao, 2006).

### MdSSK1 is a novel SKP1-like protein that is related to GSI in apple

*SSK* is a new type of SKP1-like gene though there have been reports of *SSK* genes in petunia, antirrhinum, and other plants that employ gametophytic self-incompatibility. A *SSK* gene was identified in sweet cherry, named *PavSSK1*. PavSSK1 is specifically expressed in pollen and can interact with PavSFB and PavCUL1 in yeast two-hybrid and pull-down assays (Matsumoto *et al.*, 2012). However, no SSK1-like protein has been found in *Arabidopsis* and rice, two species which do not employ *S*-RNase-based SI (Huang *et al.*, 2006; Chang *et al.*, 2009; Zhao *et al.*, 2010; Matsumoto *et al.*, 2012; Xu *et al.*, 2013). For the first time, *MdSSK1* was cloned in apple. *MdSSK1* is expressed specifically in pollen and can cluster with other SSK proteins in NJ tree analysis. Thus MdSSK1 is considered to be a pollen factor of the GSI reaction in apple.

MdSKPL1 has a structure similar to MdSSK1 and is expressed specifically in pollen, but it does not interact with other components of the SCF complex. We therefore speculate that it does not function as a bridge in the SCF complex (Tyers and Jorgensen, 2000). It appears likely that it is not the SSK1 protein that participates directly in the GSI reaction, but it is still unclear whether it can interact with other F-box that are not included in GSI, or with other genes forming the non-SCF complex (Dawson *et al.*, 2008; Jourdain *et al.*, 2009). The non-SCF complex only contains an F-box and SKP1, but nothing is known about the function of those non-SCF complexes (Hermand, 2006).

MdSKPL2 lacks some structural features of other SSK proteins, including ~140 N terminal amino acids responsible for interacting with CUL1, and the C terminal sequence which determines binding specificity with SLF is different (Schulman *et al.*, 2000; Zheng *et al.*, 2002). MdSKPL2 does not function in the SCF complex probably because of these structural differences.

### The SCF complex which contains MdSSK1 can ubiquitinate *S*-RNase

The SSK protein in pear and other plants can interact with corresponding SLF and CUL1 proteins, and it is thought to bridge these two to form the SCF complex which ubiquitinates *S*-RNase in the GSI reaction. In petunia and antirrhinum, AhSSK1 and PhSSK1 also connect with *S*-RNase via the pollen factor SLF. This proves that SSK interacts with SLF and CUL1 to form a SCF complex indirectly, although there is still a lack of direct evidence to prove the recognition between *S*-RNase and the SCF complex (Huang *et al.*, 2006; Zhao *et al.*, 2010; Matsumoto *et al.*, 2012; Xu *et al.*, 2013). It was found here that MdSSK1 can interact with S2-MdSFBB1-4 and MdCUL1, indicating that MdSSK1 is also able to bridge MdSFBB and MdCUL1 to form the SCF complex.

Next, it was examined if the SCF complex including MdSSK1 can ubiquitinate *S*-RNase *in vitro*. First, it was confirmed that *S*-RNase can be ubiquitinated in the pollen tube. Two bands with molecular masses (66kDa and 86kDa) higher than that of His-*S*_2_-RNase (approximately 26kDa) were detected in pollen tube protein treated with *S*_2_-RNase, as observed in antirrhinum (Qiao *et al.*, 2004). This suggested that *S*-RNase can be ubiquitinated in pollen tubes by some pollen factor. On the other hand, in order to exclude the possibility that *S*-RNase may adhere to the pollen tube wall (Gray *et al.*, 1999), the pollen tube was washed repeatedly to try and remove any influence of *S*-RNase on the pollen tube wall. One band with a molecular mass (about 66kDa) higher than that of *S*_2_-RNase was detected in the SCF complex reaction composed of purified MdSSK1, S2-MdSFBB1, and MdCUL1. This band may be the ubiquitinated form of *S*-RNase, and indicates that *S*-RNase can be ubiquitinated by the SCF complex *in vitro*.

One notable observation was the differing number of His-Ub (ubiquitin) labelled to *S*-RNase *in vitro* and semi-*in vivo*. Ub encodes 76 amino acids and its molecular mass is about 8kDa. The *in vitro* single band detected is 40kDa higher than *S*_2_-RNase, so there was the suspicion that the *S*_2_-RNase was labelled with 5 Ubs. While semi-*in vivo*, apart from the 66kDa band, there is also an 86kDa band, 60kDa greater than *S*_2_-RNase, which may include 7 Ubs. This might be due to the *in vitro* system inadequately simulating the natural developing environment such that *S*-RNase is incompletely ubiquitinated (Meng *et al.*, 2011). Moreover, it is in accordance with the research of others, that the denatured *S*-RNase can also be ubiquitinated *in vitro* (Yang *et al.*, 2009).

### The SCF complex containing MdSBP1 does not function in *S*-RNase ubiquitination

SBP1 in *petunia* is thought to be able to replace SKP1 in bridging F-box and CUL1 to form the SCF complex (Hua and Kao, 2006). The *S*-RNase co-incubated with PiSBP1 *in vitro* could be ubiquitinated, which indicates that PiSBP1 can function as an E3 ubiquitin ligase on *S*-RNase (Hua and Kao, 2006), but recent research indicated that PiSLF could not bind with PiSBP1 and PiSBP1 may not be part of the SCF complex (Li *et al.*, 2014). Using the apple genome, a gene homologous to *PiSBP1*, *MdSBP1*, was cloned. Firstly, *MdSBP1* was found to be expressed in pollen, style, leaves, and other organs, indicating that it might be a general protein that can function in numerous pathways. For example, NaSBP1 may be relevant to the transportation of products by endocytosis (Lee *et al.*, 2008). Secondly, MdSBP1 can interact with the hypervariable region of *S*-RNase and MdSSK1, while it does not interact with MdCUL1, MdSFBB, and MdSFBL in the yeast two-hybrid and pull-down assays. MdSBP1 can interact with the hypervariable region of *S*-RNase but is incapable of binding over the full length of *S*-RNase. The reason for this limited interaction may be irregular folding of the *S*-RNase mat protein in yeast (Sims and Ordanic, 2001). No band of ubiquitinated *S*-RNase could be detected in the reaction containing MdSBP1 or in the reaction containing MdSBP1, MdCUL1, and *S*2-MdSFBB1. MdSBP1 can bind with MdSSK1 or the hypervariable region of *S*-RNase, but it remains to be determined whether it can play a role in ubiquitinating *S*-RNase.

## Supplementary data

Supplementary data can be found at *JXB* online.

Supplementary Fig. S1. A NJ tree of plant SKP1-like proteins.

Supplementary Fig. S2. Phylogenetics analysis of the SFBB genes.

Supplementary Fig. S3. The expression patterns of MdF-box1–9.

Supplementary Fig. S4. A NJ tree of plant CUL1-like proteins.

Supplementary Fig. S5. The structure of MdSBP1 and pull-down analysis between MdSBP1 and S2-MdSFBB1–4.

Supplementary Fig. S6. Schematic diagram of *S*-RNase constructed in Y2H analysis and the interaction between non-*S*-RNase and S2-MdSFBB1-4.

Supplementary Fig. S7. The pollen germination and pollen tube length after treatment with *S*-RNase.

Supplementary Table S1. The primers used for analysis of expression patterns.

Supplementary Data
