# Supplementary material for: A novel gene, MdSSK1, as a component of the SCF complex rather than MdSBP1 can mediate the ubiquitination of S-RNase in apple
Source: J Exp Bot. 2014 Apr 23;65(12):3121–31. doi: 10.1093/jxb/eru164 (PMC4071834; doi:10.1093/jxb/eru164)
Supplement: Supplementary Data [file supp_65_12_3121__index.html]

A novel gene, MdSSK1, as a component of the SCF complex rather than MdSBP1 can mediate the ubiquitination of S-RNase in apple — A novel gene, MdSSK1, as a component of the SCF complex rather than MdSBP1 can mediate the ubiquitination of S-RNase in apple — A novel gene, MdSSK1, as a component of the SCF complex rather than MdSBP1 can mediate the ubiquitination of S-RNase in apple — Supplementary Data 

# A novel gene, *MdSSK1*, as a component of the SCF complex rather than *MdSBP1* can mediate the ubiquitination of *S*-RNase in apple

## Supplementary Data

Data files

**Files in this Data Supplement:**

- Supplementary Data - Supplementary Data
