# Supplementary material for: A novel gene, MdSSK1, as a component of the SCF complex rather than MdSBP1 can mediate the ubiquitination of S-RNase in apple
Source: J Exp Bot. 2014 Apr 23;65(12):3121–31. doi: 10.1093/jxb/eru164 (PMC4071834; doi:10.1093/jxb/eru164)
Supplement: Supplementary Data [file supp_eru164_jexbot120097_file001.pdf]

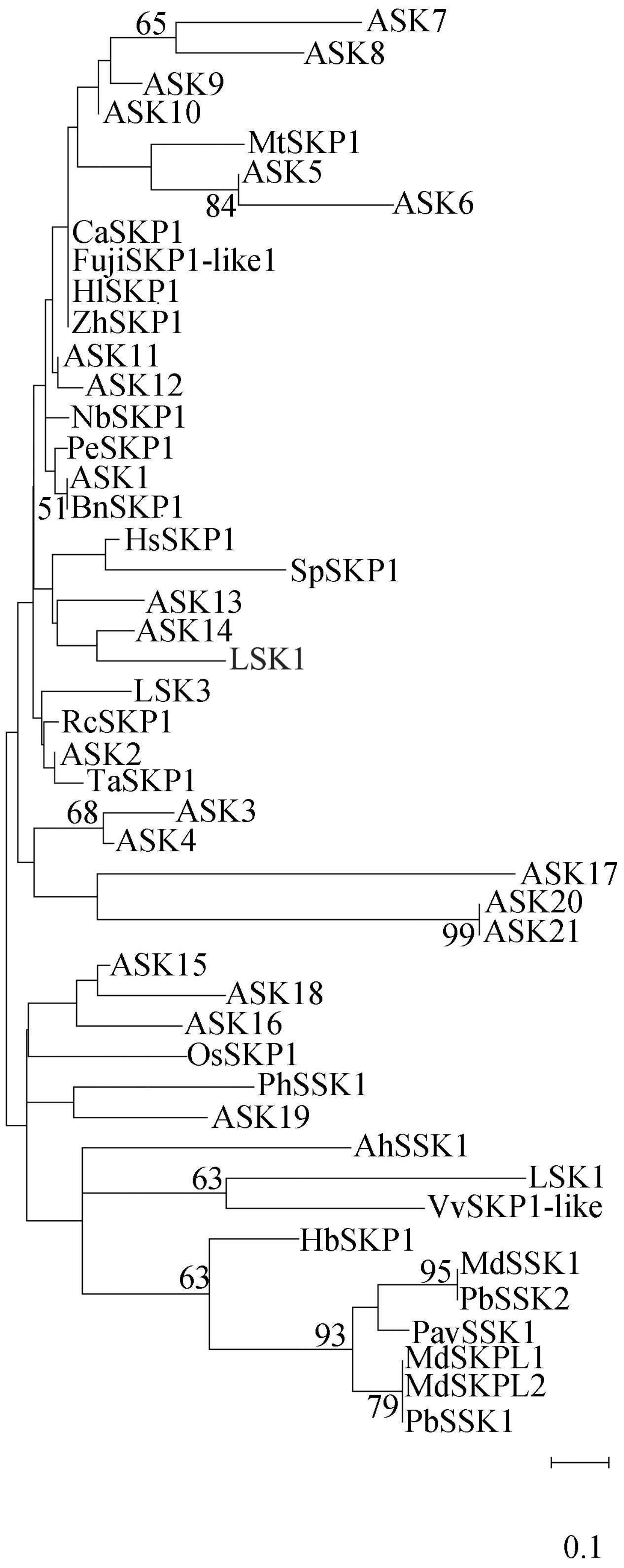

ASK7
ASK8
ASK9
ASK10
MtSKP1
ASK5
ASK6
CaSKP1
FujiSKP1-like1
HlSKP1
ZhSKP1
ASK11
ASK12
NbSKP1
PeSKP1
ASK1
BnSKP1
HsSKP1
SpSKP1
ASK13
ASK14
LSK1
LSK3
RcSKP1
ASK2
TaSKP1
ASK3
ASK4
ASK17
ASK20
ASK21
ASK15
ASK18
ASK16
OsSKP1
PhSSK1
ASK19
AhSSK1
LSK1
VvSKP1-like
HbSKP1
MdSSK1
PbSSK2
PavSSK1
MdSKPL1
MdSKPL2
PbSSK1
65
84
51
68
99
63
63
95
93
79
0.1

Supplemental Figure 1 A NJ tree of plant SKP1-like proteins. The deduced amino acid sequences were from Arabidopsis (ASK1-21), Brassica napus (BnSkp1), Zantedeschia hybrid cultivar (ZhSkp1), Humulus lupulus (HlSkp1), Capsicum annuum (CaSkp1), Fuji(FujiSkp1-like), Medicago truncatula (MtSkp1), Nicotiana benthamiana (NbSkp1), Pellia endiviifolia (PeSKP1), Triticum aestivum (TaSkp1), Oryza sativa (OsSkp1), Ricinus communis (RcSkp1), human (HsSkp1), Schizosaccharomyces pombe (SpSkp1), Petunia hybrida (PhSSK1), Antirrhinum majus (AhSSK1), Lilium longiflorum (LSK1-3), Vitis vinifera (VvSKP1-like), Hevea brasiliensis (HbSkp1), Pyrus (PbSSK1-2), Prunus (PavSSK1). The NJ tree was generated with 1,000 bootstrap replicates, and bootstrap values of 50% and above are given at branch nodes.

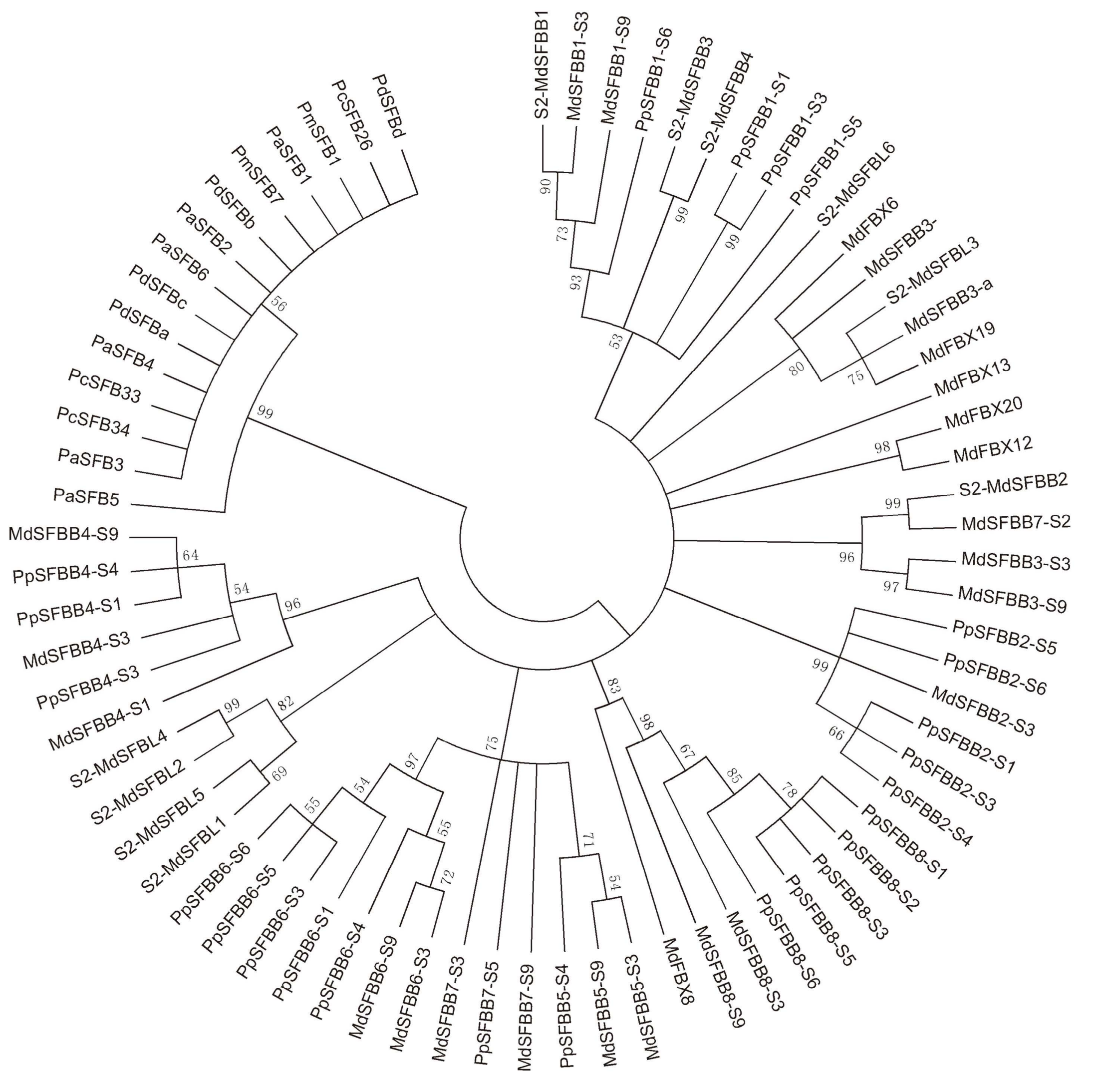

Supplemental Figure 2 Phylogenetics analysis of the SFBB genes of *Malus*, *Pyrus pyrifolia*, *P. avium, P. cerasus, P. dulcis, P. mume.* The phylogenetic tree was constructed using the Neighbor-Joining method. Numbers on the branches indicate bootstrap values >50%.

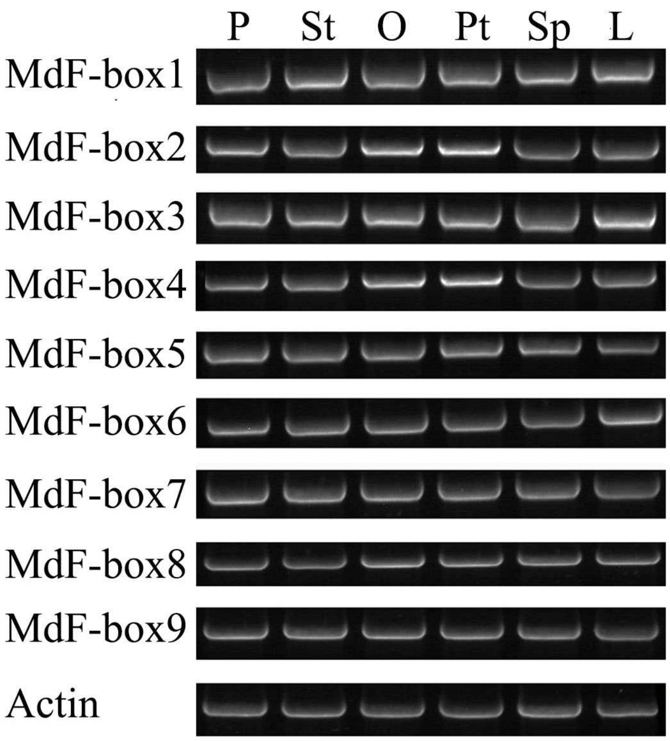

Supplemental Figure 3 The expression patterns of MdF-box1-9. Total RNAs were extracted from different organs. The synthesized cDNA were used as templates in RT-PCR. L, Leaves; Sp, sepals; Pt, petals; O, ovaries; St, styles; p, pollen.

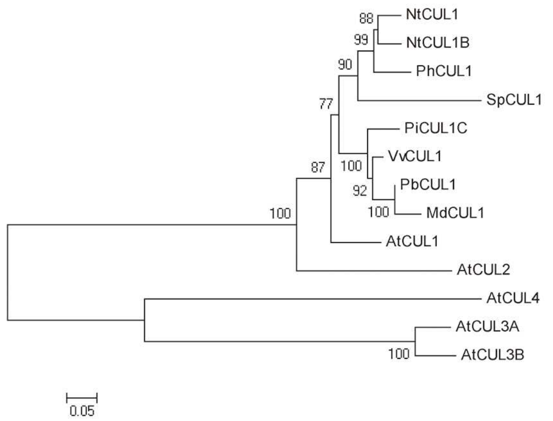

Supplemental Figure 4 A NJ tree of plant CUL1-like proteins. Arabidopsis(AtCUL1-4), tobacco(NtCUL1A-1B), tomato(SpCUL1), Vitis(VvCUL1), Petunia(PhCUL1,PiCUL1C, PiCUL1G). The NJ tree was generated with 1,000 bootstrap replicates, and bootstrap values of 50% and above are given at branch nodes.

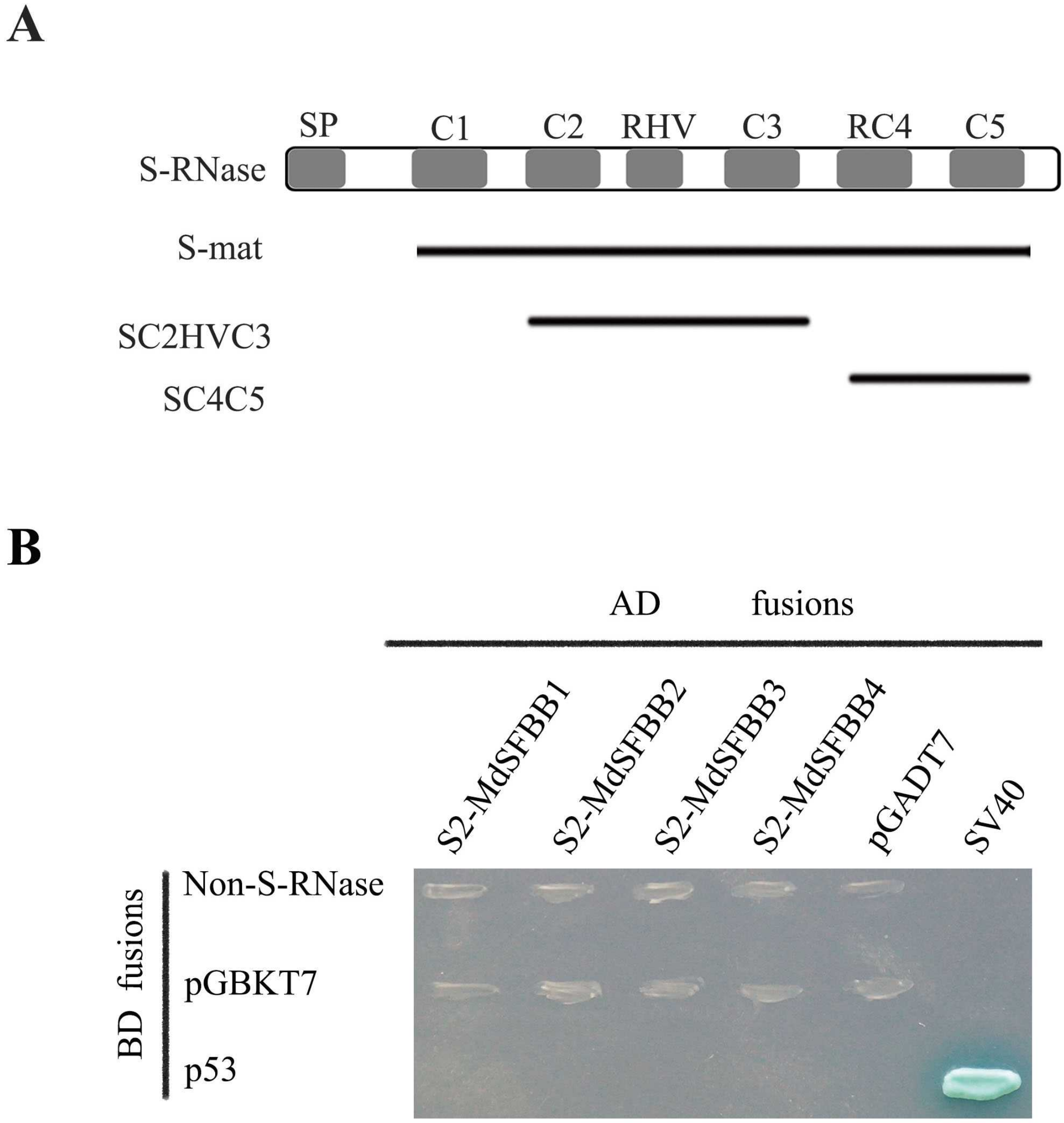

Supplemental Figure 5 Schematic diagram of S-RNase constructed in Y2H analysis and the interaction between non-S-RNase and *S2*-MdSFBB1-4. (A) C1, C2, C3, RC4 and C5 represent five conserved domains and RHV the hypervariable region, SP meaned the signal peptide in Rosaceae S-RNases. (B) Y2H assays between non-S-RNase and *S2*-MdSFBB1-4. The AD fusions and BD fusions were co-transformed into yeast strain AH109. Transformants were grown on selective medium SD/- Ade - His - Leu - Trp for 7 days at 30℃ and then were stained by X-α-gal. The p53 and SV40 were used as positive controls, the pGBKT7 and pGADT7 as negative controls.

A

```
MdSBP1 MALPQHHFQQHYPPQQQQQQQ..QQQSKSFRNLYTI.DGQLSPAVAYYDPGNLQDHPQQHPPYVPPFHVEGFAPGPVAAT  77
ScSBP1 MALPHHHLQLHI.....QQQQPHQQQSKSYRDLYNNMDGQITTPVVYFNGSNLPEQS.QHPPYIPPFQVVGLAPGTAD..  72
NaSBP1 MALPHHHLQLHS.....IQQQ..QQQSNSFRDIYNNMDGQISTPVAYFNGSNLPEQS.QHPPYIPAFQVVGLAPGPAD..  70
PiSBP1 MALPHHHLQLHI.....QQQP..QQQSKSYRDIYNNMDGQISTPVAYFNGSNLPEQS.QHPPYIPPFQVVGLAPGLVD..  70
PhSBP1 ...GTSHLQLHI.....QQQP..QQQSKSYRDIYNNMDGQISTPVAYFNCSNLPEQS.QHPPYIPPFQVVGLAPGLVD..  67

MdSBP1 DGSDNGADLQWHYGFESKRKKLKEQDFLENNSQISSIDFLQPRSVSTGLGLSLDNT.RMVSTGDSSLLSVIGDDVDHELQ  156
ScSBP1 ...DGGLDLQWNYGLEPKKKRPKEQDFMENNNSQISSVDLFQRRSVSTGLGLSLDNGRLASSCDSAFLGLVGDDIERELQ  149
NaSBP1 ...EGGLDLQWNYGLEPKKRRLKEQDFLENNNSQISSVDFLQQRSVSTGLGLSLDNGRLGSCGDSAFLGLVGDDIERELQ  147
PiSBP1 ...DGGLDLQWNYGLEPKRKRPKEQDFLENNNSQISSIDFLQPRSVSTGLGLSLDNGRLASSGDSAFLGLVGDDIERELQ  147
PhSBP1 ...DGGLDLQWNYGLEPKRKRPKEQDFLENNNSQISSIDFLQPRSVSTGLGLSLDNGRLASSGDSAFLGLVGDDIERELQ  144

MdSBP1 RQDAEIDRFLKAQGDRLRQTILDKVQATQLQTLSAVEDKVLRKLREKEAEVESISKKNMELEEQMEQLAVEAGAWQQIAR  236
ScSBP1 RQDAEIDRYIKVQGDRLRQAVLEKVQANQIQAITYVEEKVLQKLRERDTEVDDINKKNMELELRMEQLDLEANAWQQRAK  229
NaSBP1 RQDADIDRYIKVQGDRLRQAILEKVQANQLQTITCVEEKVIQKLREKEAEVEDINKKNMELELRMEQLALEANAWQQRAK  227
PiSBP1 RQDAEIDRYIKVQGDRLRQAILEKVQANQLQTVTYVEEKVIQKLREKETEVEDINKKNMELELRTEQLALEANAWQQRAK  227
PhSBP1 RQDAEIDRYIKVQGDRLRQAILEKVQANQLQTVTYVEEKVIQKLREKETEVEDINKKNMELELRTEQLALEANAWQQRAK  224

MdSBP1 RNENMISSLRFNLQHVYAQSRDSKEGCGDSEVDDTASCCNGRSINLDMFRKENNDGKEMMTCKACRVNEVCMLLLPCKHL  316
ScSBP1 YNENLINTLKVNLQHVYAQSRDSKEGCGDSEVDDTASCCNGRATDLHLLCRDSKEMKELMTCRVCRTNEVCMLLLPCKHL  309
NaSBP1 YNENLINTLKVNLQHVYAQSRDSKEGCGDSEVDDTASCCNGRATDFHLLCRDSNEMKELMTCKVCRVNEVCMLLLPCKHL  307
PiSBP1 YNENLINTLKVNLQHVYAQSRDSKEGCGDSEVDDTASCCNGRATDLHLLCRDSNEMKELMTCKVCRVNEVSMLLLPCKHL  307
PhSBP1 YNENLINTLKVNLEHVYAQSRDSKEGCGDSEVDDTASCCNGRATDLHLLCRDSNEMKELMTCKVCRVNEVSMLLLPCKHL  304
                                                                     *  *          * *

MdSBP1 CLCKDCESKLSVCPLCQSSKFIGMEVFY  344
ScSBP1 CLCKECESKLSLCPLCQSTKYIGMEVYM  337
NaSBP1 CLCKECESKLSLCPLCQSTKYIGMEVYV  335
PiSBP1 CLCKECESKLSLCPLCQSTKYIGMEIYM  335
PhSBP1 CLCKECESKLSLCPLCQSTKYIGMEIYM  332
         *  *     *  *
```

B

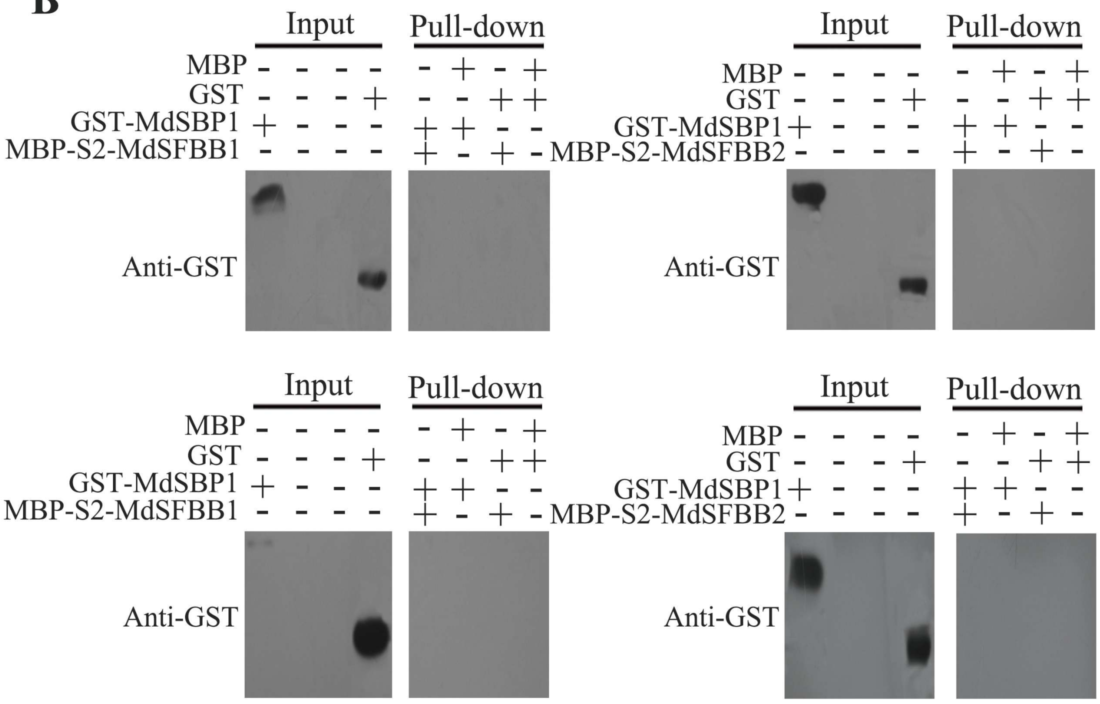

Supplemental Figure 6 The structure of MdSBP1 and pull-down analysis between MdSBP1 and S2-MdSFBB1-4. (A) Sequence alignment and structural elements of MdSBP1 and other SBP1 proteins. The pentagrams indicate the conserved cysteines. NaSBP1, PiSBP1, PhSBP1, ScSBP1, MdSBP1(from this study). (B) pull-down analysis between MdSBP1 and S2-MdSFBB1-4. Purified S2-MdSFBB1-4 were used as bait against purified GST-MdSBP1. Bound proteins were examined with anti-GST

antibody. GST and MBP were used as negative controls.

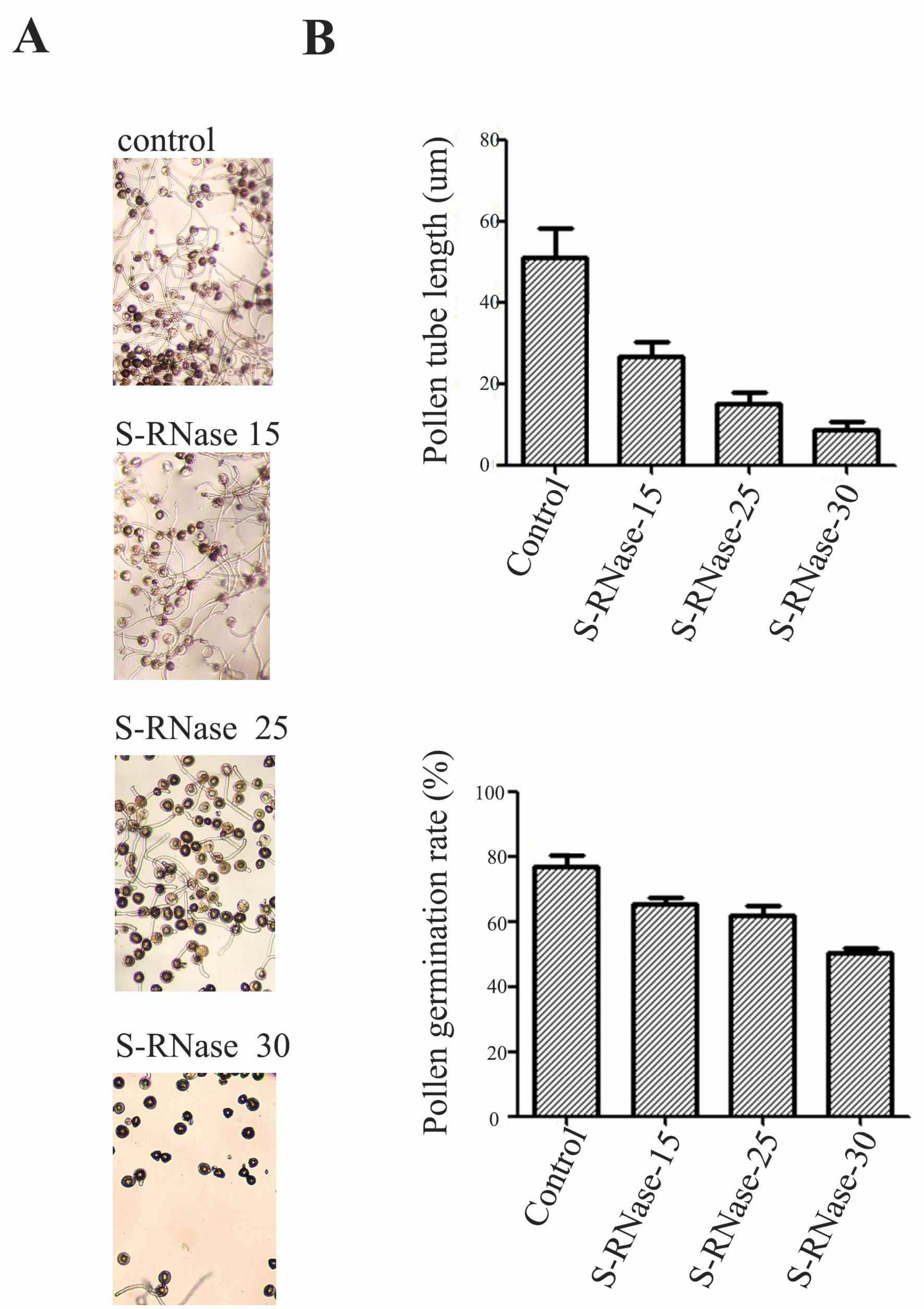

Supplemental Figure 7 The pollen germination and pollen tube length after treated with *S*-RNase. (A) Pollen growth status with different concertration S-RNase treated. (B) The pollen germination rate (3 replicated with 100-pollen grains, $p<0.01$) and

pollen tube length (3 replicated with 100-pollen grains, $p<0.01$) under different treatment by S-RNase.

The Primers used for analysis of expression pattern

Primer	Sequence
S2-MdSFBB1-F	ATAGCAGGGACAAGTCTTTATTTGATAA
S2-MdSFBB1-R	AAAATCTCTA ACTTCATTG GAATA
S2-MdSFBB2-F	AATCTTTCCATTGATAGTGATGTACAT
S2-MdSFBB2-R	GAAAAGCCCAAAACTAAAGCCAACT
S2-MdSFBB3-F	CTTCATTTGATAAATGTTCTT
S2-MdSFBB3-R	GGATTCACCCTTCGAAATGTTG
S2-MdSFBB4-F	CAGTCGCAAGATCAAGAAGT
S2-MdSFBB4-R	CTAAATCAAATGAAAGTATGTATT
S2-MdSFBL1-F	ATGGAATACCATCATCCTGTATTGATTC
S2-MdSFBL1-R	TGACTGATTCATTATACAGA AAAAGA
S2-MdSFBL2-F	GTTCTTGAATTGTTCGGAATTAAC
S2-MdSFBL2-R	ATTCCTCCAA GATCCACTTCTTT
S2-MdSFBL3-F	TGTGTGTCAAAGTGAATCTCTA
S2-MdSFBL3-R	TATGAACAATACTTTTTACATAA
S2-MdSFBL4-F	TGGAAGACCATCATCCTGTAGT
S2-MdSFBL4-R	TGAGAAAGGAGGTGGAGAACG
S2-MdSFBL5-F	ACCCCTGCAGATAAGGCGGTCGAA
S2-MdSFBL5-R	TTACTTGATAGGAACAATAGTTTCC
S2-MdSFBL6-F	GGTGCATGAAAATGAAACTCTT
S2-MdSFBL6-R	ATGAAACAATACTTTTCACATAC

Supplemental Table 1
